# Efficacy and Functional Mechanisms of a Two-Stage Pretreatment Approach Based on Alkali and Ionic Liquid for Bioconversion of Waste Medium-Density Fiberboard

**DOI:** 10.3390/molecules29092153

**Published:** 2024-05-06

**Authors:** Shujie Wang, Xianfeng Hou, Jin Sun, Dan Sun, Zhenzhong Gao

**Affiliations:** College of Material and Energy, South China Agricultural University, Guangzhou 510642, China; sjwang@stu.scau.edu.cn (S.W.); xfhou@scau.edu.cn (X.H.); sunjin2003@163.com (J.S.); 13931925960@163.com (D.S.)

**Keywords:** medium-density fiberboard, pretreatment, enzymatic saccharification

## Abstract

A novel pretreatment strategy utilizing a combination of NaOH and 1-Butyl-3-methylimidazolium chloride ([Bmim]Cl) was proposed to enhance the enzymatic hydrolysis of abandoned Medium-density fiberboard (MDF). The synergistic effect of NaOH and [Bmim]Cl pretreatment significantly improved the glucose yield, reaching 445.8 mg/g within 72 h, which was 5.04 times higher than that of the untreated samples. The working mechanism was elucidated according to chemical composition, as well as FTIR, ^13^C NMR, XRD, and SEM analyses. The combined effects of NaOH and [Bmim]Cl led to lignin degradation, hemicellulose removal, the destruction and erosion of crystalline regions, pores, and an irregular microscopic morphology. In addition, by comparing the enzymatic hydrolysis sugar yield and elemental nitrogen content of untreated MDF samples, eucalyptus, and hot mill fibers (HMF), it was demonstrated that the presence of adhesives and additives in waste MDF significantly influences its hydrolysis process. The sugar yield of untreated MDF samples (88.5 mg/g) was compared with those subjected to hydrothermal pretreatment (183.2 mg/g), Ionic liquid (IL) pretreatment (406.1 mg/g), and microwave-assisted ionic liquid pretreatment (MWI) (281.3 mg/g). A long water bath pretreatment can reduce the effect of adhesives and additives on the enzymatic hydrolysis of waste MDF. The sugar yield produced by the combined pretreatment proposed in this study and the removal ability of adhesives and additives highlight the great potential of our pretreatment technology in the recycling of waste fiberboard.

## 1. Introduction

Current worldwide energy consumption is mainly dependent on fossil fuels [1,2]. However, the progressive depletion of fossil fuel sources, the environmental problems caused by them, recent unstable fuel prices, and increasing political pressures have driven researchers develop sustainable, environmentally friendly, and renewable fuel resources [3,4]. One of the most promising alternatives to fossil fuels is lignocellulosic biomass [5,6]. Meanwhile, global concerns over the accumulation of waste wood-based panel residues have arisen. For instance, according to the China Forestry Development Report, approximately 10% of wood-based panel materials and their related products are discarded every year in China, with an approximate amount of 30 million m^3^ in 2022 [7]. MDF is mainly composed of hot-milled fibers (mainly composed of cellulose, hemicellulose, lignin, about 85% of the mass of fiberboard), adhesives (urea-formaldehyde resin (UF) or phenolic resin (PF), about 8–12% of the mass of fiberboard), and waterproofing agents (paraffin, rosin, about 1–2.5% of the mass of fiberboard), as well as adhesive curing agents (acidic substances, resin mass 0.5–2%), adhesive fillers (starch, soybean powder, resin mass 5–50%), flame retardants (phosphorus-nitrogen flame retardants, boron flame retardants), preservatives (sodium pentachlorophenol, alkylammonium), and other additives [8]. At present, the main treatment method used for waste wood-based panels in China is burial or incineration, which will not only waste natural resources such as timber, but also worsen the negative impacts on ecological resources such as air, soil, and groundwater. If waste wood-based panel furniture is recycled or remanufactured into new products, it will help to save resources and energy [7]. The conversion of waste wood-based panels into fermentable sugars would not only contribute to the appropriate disposal of waste wood-based panel materials, but would also be favorable for fuel generation and alleviating the energy crisis.

As a renewable, carbon-neutral material, lignocellulosic biomass can be converted into biofuel and other intermediate chemicals via different conversion routes [9]. However, lignocellulosic biomass processing can be costly because hemicellulose binds to cellulose microfilaments, lignin, and pectin forming a cross-linked network, which fills the microfibrils of the secondary plant cell wall and prevents enzymes and chemicals from accessing the hydrophilic cellulose chains and breaking β-(1,4)-glycosidic bonds between monomeric units of glucose. Moreover, lignin has strong adsorption properties for biological enzymes and cellulose with a high degree of polymerization and crystallinity makes the cellulose chain more difficult to be cut by enzyme molecules, thereby preventing cellulose from degradation by cellulolytic enzymes [10]. Pretreatment of lignocellulosic biomass is required to alter the physical and chemical structure of plant cell walls to facilitate the removal or redistribution of cell wall components, thereby increasing the area of carbohydrates that are accessible to cellulolytic enzymes [11,12]. Moreover, the adhesives and additives present in MDF tend to envelop the surface of dried fibers, thereby reducing cellulose accessibility. Additionally, the urea-formaldehyde or phenolic resins and additives used in MDF possess a certain chemical toxicity towards cellulose hydrolase, which may impede the efficiency of enzymatic hydrolysis. Moreover, it is also important to understand what effects pretreatment degradation can have upon process completion.

Because lignocellulose is resistant, the majority of the sugars it contains must be released during a pretreatment process. Various pretreatment techniques such as steam explosion, concentrated acid, dilute acid, hot water, organosolv, and ionic liquid pretreatments are currently being developed [13,14]. The application of ILs in biomass pretreatment is increasingly considered as a novel, efficient, and environmentally friendly biomass deconstruction method. Due to their ‘green’ properties, such as non-volatility, non-flammability, high chemical and thermal stability, wide electrochemical range, high ionic conductivity, recyclability, and wide solubility, ILs have attracted significant attention as potential biomass pretreatment agents [15,16]. Although using ILs as a pretreatment agent has many advantages over traditional methods, it is costly, and their relative effectiveness in removing lignin and hemicellulose hinders their wide application. Sodium hydroxide will induce the cross-linking of hemicellulose through easter bonds and saponify the lignin component. Saponification leads to the cleavage of lignin–carbohydrate complex bonds, and the exposure of the cellulose microfibrils can increase the enzymatic digestibility of cellulose [15]. Acetyl and various uronic acid substitutes were also removed by alkali, thereby reducing the steric hindrance of the hydrolase, increasing the accessibility of carbohydrates to the enzyme, and facilitating subsequent enzymatic hydrolysis [17]. At the same time, the hydrolysis reaction of UF resin residues is the reverse reaction of its synthesis process, and the water molecules penetrate the junction of UF resin to initiate the hydrolysis reaction. Due to the presence of hydroxymethyl and methylene ether bonds in UF resin, UF resin residues are more likely to hydrolyze under hydrothermal conditions [18]. Therefore, the sodium hydroxide solution is the preferred target for the pretreatment of waste MDF. However, independent alkali pretreatment has limitations such as a narrow matrix range and poor pretreatment effects. Therefore, in order to overcome the limitations of IL or alkali alone, a combined pretreatment method can be developed, which can be skilled, environmentally friendly, and superior to traditional techniques. A two-step pretreatment process with a mild alkali and ionic liquids more effectively improves the accessibility of cellulose than a one-step pretreatment with a strong alkali or ionic liquids [19,20]. Pretreatment helps to degrade adhesives and additives, remove hemicellulose and lignin, and improve the efficiency of sugar conversion, so that more wooden industrial wastes can be digested.

In this study, a series of pretreatment designs and experimental results have been reported to evaluate the effect of pretreatment on MDF powders. The content of each component in MDF powder was determined, and the enzymatic hydrolysis sugar yield of fiberboard samples under different pretreatment conditions was determined. Fourier-transform infrared (FT-IR) spectroscopy, X-ray diffraction (XRD), ^13^C nuclear magnetic resonance (^13^C NMR), scanning electron microscopy (SEM), and elemental analysis (EA) were implemented to elucidate the reaction mechanism of the different pretreatments examined. To the best of our knowledge, waste MDF was used here for the first time as a material for biomass energy conversion in our study. Additionally, the influence of additives and their degradation on its hydrolysis parameters has been discussed in the present paper. These results provide a valuable reference for the development of a novel technology for the pretreatment of scrap MDF so that it can be recycled.

## 2. Results and Discussion

### 2.1. Determination of the Optimal Pretreatment Process

#### 2.1.1. Effect of NaOH Pretreatment on the Enzymatic Hydrolysis of MDF

The enzymatic hydrolysis of MDF samples, which were pretreated with varying concentrations of NaOH solution, was conducted in a constant temperature water bath at 90 °C for different durations and the results are summarized in Table 1. The sugar yields from the MDF samples following 1 h pretreatment with 1%, 3%, and 5% NaOH solution for 72 h were 350.28 mg/g, 360 mg/g, and 378.27 mg/g, respectively. The sugar yield decreased with the drop in NaOH concentration. The relationship between the sugar yield and NaOH concentration showed the same trend at pretreatment durations of 1.5 and 2 h. Moreover, at a constant concentration of the NaOH solution, the increase in the pretreatment time correspondingly led to a decrease in the sugar yield, which was related to the degradation of holocellulose caused by too severe pretreatment conditions [21]. with the best results being obtained following the 1 h pretreatment with 5% NaOH solution. Therefore, a pretreatment with 5% NaOH aqueous solution at 90 °C for 1 h is the best choice for the subsequent two-step pretreatment.

#### 2.1.2. Impact of AAI Pretreatment on MDF Enzymatic Hydrolysis

**Figure 1:**
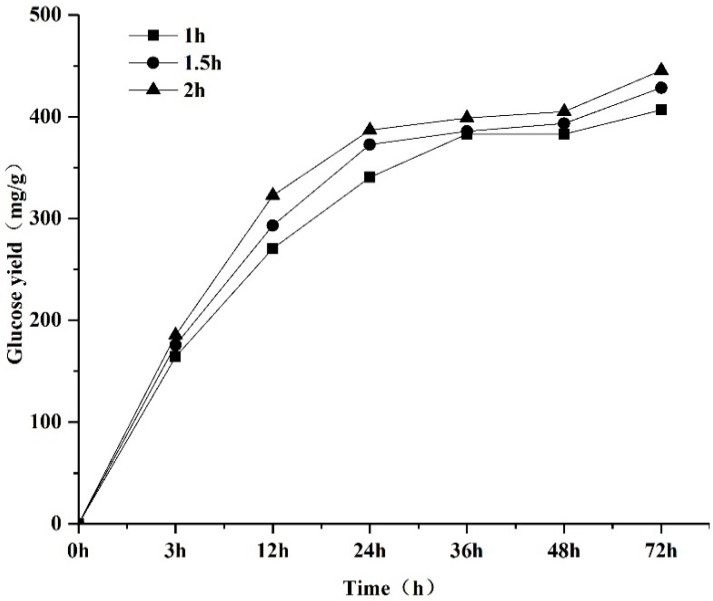
Effect of pretreatment time of AAI on glucose yield.

Pretreatment of the samples with the NaOH solution with a concentration of 5% for 1 h was the first step, and IL pretreatment for different durations was the second step. As can be seen in Figure 1, 72 h after the enzymatic hydrolysis, the final glucose yield from the MDF after 1 h AAI pretreatment was 406.8 mg/g; then, the pretreatment time was extended to 1.5 h, which led to an increase in the glucose yield from MDF conversion to 428.4 mg/g. The prolongation of the pretreatment time to 2 h resulted in the highest glucose yield of 445.5 mg/g. A series of studies were conducted to explore the potential mechanism of AAI pretreatment, as described below.

### 2.2. Assessment of the Potential of Disused MDF Additive to Affect the Enzymatic Hydrolysis Sugar Yield

**Figure 2:**
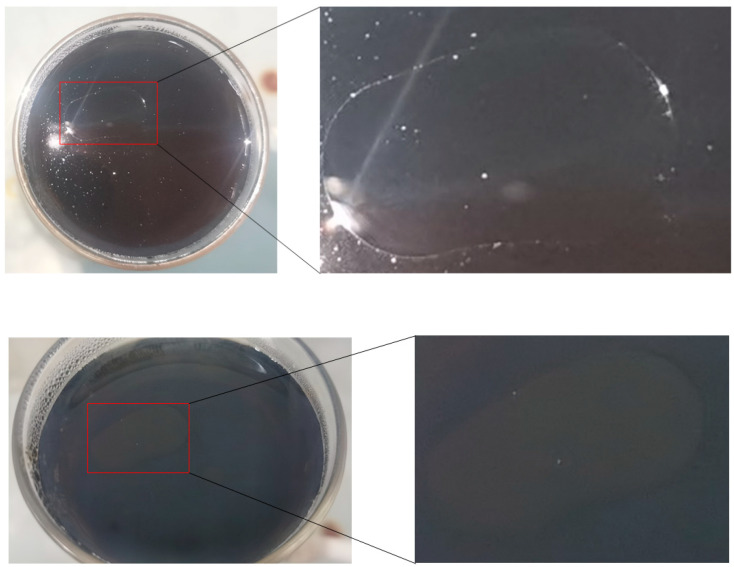
The oily substance in the sand core funnel (10 g MDF sample).

After NaOH pretreatment, oily substances were detected on the liquid surface of the sintered glass filter after the separation of the MDF powder from the NaOH solution (Figure 2). The same pretreatment for preparing MDF hot-mill fiber (HMF) did not yield such oily substances. According to the production process for fiberboard, it is speculated that the observed oily substances may be composed of a waterproof agent (paraffin, rosin) or an oily flame retardant or preservative.

After the cellulase hydrolysis of the eucalyptus, HMF, and MDF powders for 72 h, experiments were conducted to investigate whether the oil substances impact the enzymatic hydrolysis of MDF. According to Table 2, the sugar yields from these samples were 193.2 mg/g, 216.1 mg/g, and 88.5 mg/g, respectively. The higher yield from the HMF is attributed to the partial decomposition of the eucalyptus cell wall during the heat milling process, surpassing that of the eucalyptus powder. However, the sugar yield from the MDF powder was notably lower than those from the eucalyptus and HMF, largely due to the adhesives and additives used during MDF production which bind the HMF into a board.

Further experiments involved soaking the eucalyptus, HMF, and MDF powders in hot water at 90 °C for one hour before they underwent 72 h of cellulase hydrolysis; the results are documented in Table 2. Post-treatment, the sugar yields were measured at 205.4 mg/g, 248.8 mg/g, and 167.6 mg/g, respectively. It is noteworthy that while the sugar yields from the eucalyptus and HMF remained relatively stable, the yield from the MDF powder increased significantly by 1.89-fold compared to the untreated MDF. This increase could likely be due to the effective removal of the oily substances during the hydrothermal pretreatment.

Additionally, the impact of long-term hydrothermal pretreatment on eliminating adhesives and additives in MDF was evaluated, and the results displayed in Table 3. The elemental nitrogen content in the eucalyptus sample was 0.95%, but in the MDF sample, it was significantly higher at 4.60%—approximately 4.84 times the amount found in eucalyptus—which was likely due to the addition of urea-formaldehyde resin and other additives during MDF production. After hydrothermal pretreatment, the nitrogen content in the MDF sample decreased significantly, while the sugar content increased considerably, indicating that extended hot water pretreatment can effectively remove adhesives and additives from MDF. The sugar yield from MDF after MWL pretreatment was 281.3 mg/g, whereas after BW + IL pretreatment, it rose to 406.1 mg/g, surpassing the yield after MWL pretreatment. These findings differ from those reported in Hou’s study [22], which suggested that the nitrogen content after BW + IL pretreatment was lower than that after MWL pretreatment, thus supporting the necessity of a longer hydrothermal pretreatment to effectively remove adhesives and additives in MDF. Our data clearly indicate that the presence of additives and adhesives in MDF significantly affects the sugar yield, underscoring the importance of their removal for optimizing enzymatic hydrolysis outcomes.

### 2.3. Component MDF Content under Different Pretreatment Conditions

The quantification and monitoring of the lignocellulosic biomass components—cellulose, hemicellulose, and lignin—are crucial for accurately evaluating the impact of various pretreatments. Table 4 details the chemical composition of untreated MDF samples compared to those treated with NaOH and AAI. Initially, the glucan content in untreated MDF was 38.18%, with xylan at 27.45%, and lignin at 27.35%. Post NaOH pretreatment, the glucan content increased to 48.91%, while the xylan and lignin levels decreased to 18.93% and 23.67%, respectively. This aligns with prior studies indicating that enhanced enzymatic hydrolysis of MDF following NaOH pretreatment results from the removal of hemicellulose and lignin [23,24]. After AAI pretreatment, the analysis determined the contents of glucan, xylan, arabinose, and lignin in the MDF samples. The cellulose content significantly increased to 53.4%, while the hemicellulose and lignin contents were reduced to 16.14% and 21.4%, respectively. The AAI pretreatment demonstrated a superior capacity to remove hemicellulose and lignin compared to the NaOH pretreatment, which likely contributes to the observed higher yields of reducing sugars in the fiberboard. Furthermore, subsequent NaOH pretreatment markedly enhanced the yields of cellulose and hemicelluloses from their initial levels of 23.28% and 27.25% to 77.34% and 43.21%, respectively. Following the AAI pretreatment, these yields further increased to 83.42% and 51.74%. This two-step pretreatment strategy has proven to be effective in accelerating the degradation rate of lignin and hemicellulose, thereby improving the glucose conversion efficiency significantly.

### 2.4. Chemical Structure Analysis

**Figure 3:**
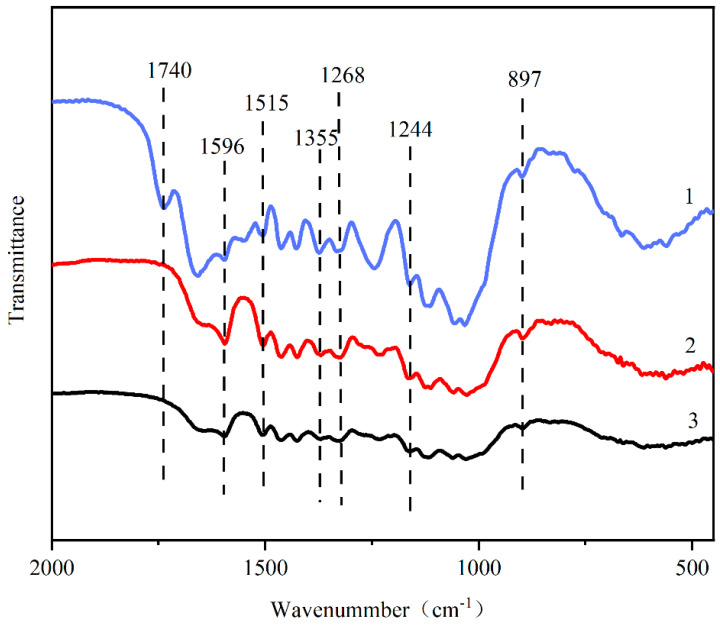
FT-IR spectra of the raw MDF, NaOH-pretreated, and AAI-pretreated MDF: 1—untreated MDF; 2—NaOH pretreated; 3—AAI pretreated.

FT-IR is extensively utilized to examine the changes in chemical functional groups during pretreatments. Figure 3 and Table 5 present the FT-IR spectra of the pretreated solids and attribute characteristic peaks to specific functional groups, respectively. Notably, the band at 897 cm^−1^ suggests the disruption of the β-(1,4)-glycosidic bond (C-O-C) between cellulose and hemicellulose [25,26]. This observation underlines the efficacy of NaOH and AAI pretreatments in removing lignin and hemicellulose, thus facilitating enhanced interactions between cellulose and enzymes. In the spectra of untreated raw materials, absorption peaks at 1740 and 1268 cm^−1^ are observed. These are traditionally associated with the C-O stretch of the acetyl group and an ester stretching vibration in hemicellulose, respectively [27,28,29]. Post-treatment, these bands were significantly diminished or absent, indicating the substantial removal of the acetyl group and hemicellulose during the pretreatment process, a finding corroborated by our compositional analysis (Table 4). Furthermore, there were significant alterations in the band intensities at 1515, 1596, and 1355 cm^−1^, which are linked to the C-H out-of-plane vibration and aromatic skeletal vibrations of lignin [30,31]. The pretreated solids exhibited reduced signals at these bands compared to untreated MDF, with notably weaker band intensities in NaOH- and AAI-pretreated solids due to the reduced lignin content. These results highlight the NaOH pretreatment’s robust and effective delignification mechanism.

**Figure 4:**
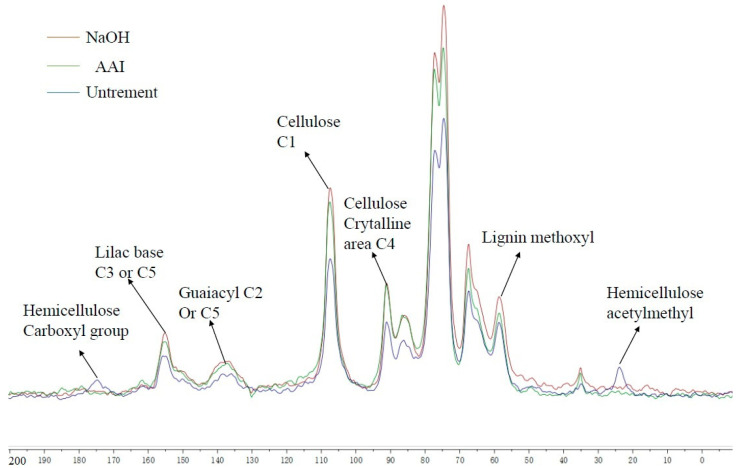
^13^C NMR spectra of raw MDF and NaOH- and AAI-pretreated MDF.

The ^13^C NMR spectra of untreated and NaOH- and AAI-pretreated MDF were divided into three main regions: the first region was the vibrational absorption peak of the carbon atom belonging to the lignin aromatic ring, shifting from 160 to 120 ppm. The second region was the shift from 96 to 60 ppm, representing the vibrational absorption peak of the carbon atom on the structural carbohydrate [32]. The third region was the vibrational absorption peak of the carbon atoms in the fatty carbon chain, ranging from 50 to 20 ppm. Meanwhile, as can be seen in Figure 4, some differences were observed between the ^13^C NMR spectra of the unprocessed MDF and the MDF after pretreatment with NaOH and AAI. The absorption peaks at 21 and 172 ppm were attributed to the carbon atoms in hemicellulose, mainly to the vibration of the carbon atoms in the sugar ring [33]. The absorption peaks of the untreated MDF samples were clear at these two wavelengths, whereas the characteristic absorption peaks of hemicellulose were significantly weakened after the NaOH and AAI pretreatments. This showed that hemicellulose had been removed or destroyed during the pretreatment. The characteristic absorption peak of AAI pretreatment was lower than that of NaOH pretreatment, indicating that AAI pretreatment could degrade more cellulose. This result was consistent with the ones for previous sample composition and infrared analyses, where the degradation of hemicellulose was positively correlated with the enzymatic hydrolysis of the sample [34]. The absorption peaks at 133.6 and 153 ppm were the absorption peaks of the aromatic carbon atom, whereas the absorption peaks at 153 ppm represented the absorption peaks of the third and fifth carbon atoms of the eugenyl group [35]. The vibration of NaOH- and AAI-pretreated MDF at the peaks of 133.6 and 153 ppm, respectively, was significantly lower than that of untreated MDF. Therefore, the pretreatment with NaOH and AAI removed certain amounts of lignin from the MDF structure, which was confirmed by the results of the lignin content determination in the analysis of the main components before and after the MDF pretreatment. The absorption peaks at 73.2 and 71.2 ppm were the absorption peaks of the two, three, and five carbon atoms on the cellulose furan ring. Additionally, the absorption peak at 104.8 ppm was attributed to the absorption vibrational peak of the carbon atom at position 1 of cellulose [36]. The absorption peak at 85.6 ppm was the vibrational absorption peak of the carbon atom at the fourth position of the cellulose in the crystalline region [37]. Compared with untreated MDF, the signal strength of cellulosic C in pretreated MDF was significantly higher, indicating that NaOH and AAI pretreatment increased the relative cellulose content in MDF, which was consistent with the higher lignin content established by the component analysis and the analysis of the FT-IR experimental results. The increase in the relative signal strength at the chemical shift of 85.6 ppm indicates that the relative proportion of amorphous cellulose in MDF was also elevated after the pretreatment. It is possible that the removal or dissolution of lignin or hemicellulose led to an augmentation in the relative content of amorphous cellulose.

### 2.5. Crystal Index Analysis

**Figure 5:**
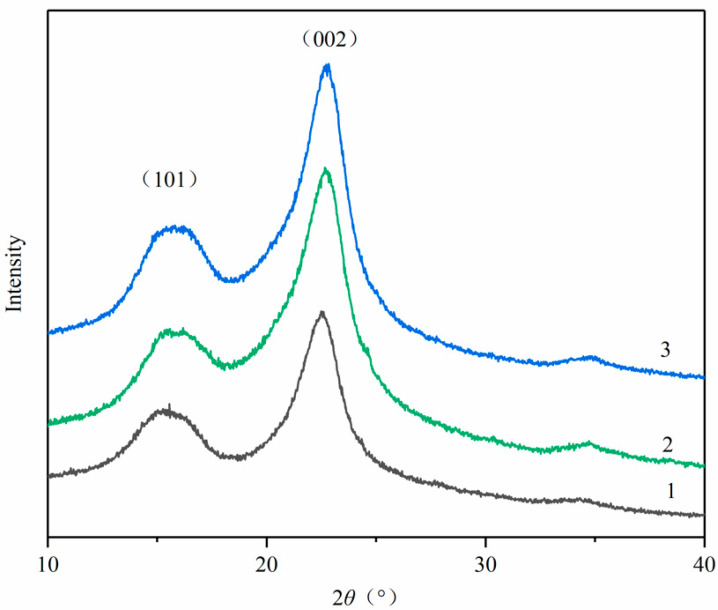
XRD diffractograms of the raw MDF, NaOH-pretreated, and AAI-pretreated MDF; 1—untreated MDF; 2—NaOH pretreated; 3—AAI pretreated.

The efficiency of enzymatic sugar hydrolysis is influenced by the cellulose Crystallinity Index (CrI), which was quantified using XRD. Figure 5 illustrates a graph comparing the diffraction patterns between untreated biomass and biomass subjected to AAI and NaOH pretreatments. Variations in the position of the diffracting peaks highlight the shifts in the distances between hydrogen-bonded cellulose sheets. Notably, the two peaks at 2θ of 22.5° and 18.5° correspond to Cellulose-I and -II, respectively. The prominent peak at 22.5° represents a highly organized crystalline region (Iα and Iβ), while the smaller peak at 18° denotes the less organized region (Cellulose-II). According to formula (1), the pretreatments did not introduce new peaks, thus indicating no significant alteration in the crystal morphology of cellulose. The CrI of untreated MDF was recorded at 47.59%, which rose to 61.35% and 63.83% after NaOH and AAI pretreatments, respectively. This increase is plausible as both lignin and hemicellulose, which are inherently amorphous, undergo partial removal during pretreatment. Other research supports that a lower proportion of hemicelluloses or a reduced non-crystalline cellulose region can facilitate more effective enzymatic sugar hydrolysis, associated with an enhanced CrI [38,39,40].

### 2.6. Microstructure Analysis

**Figure 6:**
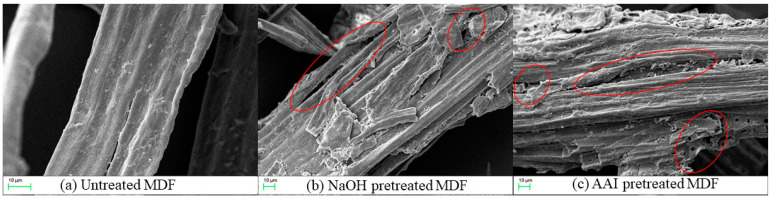
SEM images of (**a**) untreated MDF; (**b**) NaOH-pretreated MDF; (**c**) AAI-pretreated MDF.

SEM was employed to monitor the morphological alterations in the biomass resulting from the various NaOH and AAI pretreatments. The untreated substrate displayed a rigid and smooth surface morphology as depicted in Figure 6a, which impedes the accessibility of cellulase enzymes to the cellulose [10,41]. The NaOH and AAI pretreatments lead to the formation of a porous and rough surface on MDF (red circle in the picture), suggesting that the pretreatment disrupts the cellulose–hemicellulose–lignin matrix and removes some external fibers. This alteration enhances the enzymes’ accessibility to cellulose, thereby increasing the sugar yield from enzymatic hydrolysis [42,43]. In contrast, Figure 6b shows that MDF surfaces treated solely with NaOH exhibit partial deformation and structural disintegration, potentially showing lignin hydrolysis, which serves as the structural backbone for microfibril integrity. Following AAI pretreatment, the cell wall displays irregularities and roughness, as seen in Figure 6c, with loosened fiber networks within the pulp, increased porosity, and the emergence of surface cracks, thus significantly revealing the internal structure and composition of the biomass. These observations are consistent with the results from the chemical composition analysis in Table 4, which indicate a reduction in lignin content after the combined pretreatment.

## 3. Materials and Methods

### 3.1. Materials

MDF obtained from the quality supervision and inspection station of wood and wood products in Guangdong province was crushed with a grinder, and 40–80 mesh powder (0.18–0.425 mm) was separated through a screen. All chemicals are reagent grade, and purchased from Damao chemical reagent factory unless otherwise stated. ([Bmim]Cl was purchased from D&B with a 99% purity, and cellulase and β-glucosidase were purchased from Ruiyang. The enzyme activity was between 50,000 u/g and 300 u/g). All raw materials were oven-dried overnight in a high temperature of 103 °C to eliminate moisture content change and the need for further preprocessing.

### 3.2. Pretreatment

#### 3.2.1. NaOH Pretreatment

MDF samples weighing 1.0 g underwent pretreatment at a temperature of 90 °C by immersing them in NaOH solutions with concentrations of 1 wt%, 3 wt%, and 5 wt% for durations of one hour, one and a half hours, and two hours, respectively; the volume used was set at precisely 33.3 mL. Following pretreatment, distilled water was used to wash the biomass samples until they were neutral. The recovered particles were heated to 103 °C in a draught dryer for 12 h before being sealed in a plastic bag.

#### 3.2.2. NaOH + IL Pretreatment (AAI Pretreatment)

The MDF sample (1.0 g) that showed the best NaOH pretreatment effect was mixed with [Bmim]Cl (10 g) at 90 °C. After pretreatment for 1, 1.5, and 2 h, respectively, the biological sample was washed with hot distilled water for neutrality. The recovered solids were baked in a 103 °C draught dryer stove for 12 h and then stored in a sealed plastic bag.

#### 3.2.3. Hydrothermal Pretreatment

The MDF samples (1.0 g) were mixed at 90 °C with 33.3 mL of water for two hours. Following pretreatment, distilled water was used to wash the biomass samples until they were neutral. The recovered solids were dried for 12 h at 103 °C in a draught drier before being placed in a sealed plastic bag.

#### 3.2.4. Microwave + Ionic Liquid Pretreatment

The MDF sample (1.0 g) was mixed with [Bmim]Cl (10 g), and placed in the microwave at medium-high for 6 min. The biological sample was washed with distilled water. The recovered solid was baked for 12 h at 103 °C in a draught dryer stove before being placed in a sealed plastic bag for storage.

### 3.3. Composition Analysis

The mass fractions of glucan, xylan, arabinose, and lignin (the combined content of acid-soluble lignin and acid-insoluble lignin) in untreated MDF and pretreated MDF residues were determined using the standard methodology established by the National Renewable Energy Laboratory (NREL) [44].

### 3.4. Enzymatic Hydrolysis of Cellulose

A pretreated 0.05 g sample was placed in a 50 mL Erlenmeyer flask along with 35 FPU/g of cellulase and 20 mL of a citric acid buffer with a pH of 4.8. The sealed conical flask was then shaken for 72 h at a temperature of 50 °C at a speed of 150 rpm on a constant temperature shaker. The 3,5-Dinitrosalicylic acid (DNS) test method was used to determine the presence of reducing sugars. The concentration of fermentable sugars such as glucose and xylose in the liquid was determined by HPLC [45].

### 3.5. Physicochemical Characterization of Pretreated MDF Biomass

The ultrastructure of MDF was investigated by employing Fourier Transform Infrared Spectroscopy (FTIR), ^13^C Nuclear Magnetic Resonance Spectra (^13^C NMR), X-ray Diffractometer (XRD), Scanning Electron Microscopy (SEM) analysis, and Elemental Analysis (EA) to assess the effects of NaOH pretreatment and AAI pretreatment.

#### 3.5.1. FTIR

An FTIR spectrophotometer recorded the FTIR spectrum (NICOLET6700, Thermo, Waltham, MA, USA). To prepare the samples (KBr pellets) for analysis, 1 mg of material powder was combined with 100 mg of KBr. A total of 32 scans were performed between 4000 and 400 cm^−1^.

#### 3.5.2. ^13^C NMR

Using a Bruker AV-III 400 M spectrometer (Avance; Bruker, Billerica, MA, USA), solid-state cross-polarization/magic angle spinning (CP/MAS) ^13^C NMR spectra of cellulose fractions were acquired at 100.6 Hz. Using a CP pulse program with a 1 ms match time and a 2 s delay between transients, measurements were carried out on dry cellulose fractions packed onto a 4 mm zirconia (ZrO_2_) rotor. At a spinning rate of 5 kHz, a total of 5000 scans were recorded.

#### 3.5.3. XRD

Both pretreated and untreated specimens underwent XRD analysis on a D8 ADVANCE diffractometer with a sealed-tube Cu-Kα source. The X-ray wavelength was 0.15406 nm, and the working voltage and current were 40 kV and 40 mA, respectively. With a step size of 0.03 and a time interval of 4 s, scans were taken from 2θ = 10 to 50°. The crystallinity index was defined as follows [46]:

(1) $$\mathrm{CrI}=\frac{I_{002}-I_{\mathrm{am}}}{I_{002}}\times 100\%$$

where I_002_ is the crystalline peak of the maximum intensity at 2θ between 22° and 23° and I_am_ is the minimum intensity at 2θ between 18° and 19°

#### 3.5.4. SEM

Scanning electron microscopy (SEM) analysis of the untreated and regenerated Cellulose was carried out with a Hitachi S-3400N II (Hitachi, Tokyo Metropolis, Japan) instrument at 5 kV. The samples were sputter-coated with a thin layer of gold prior to taking pictures. All images were taken at 500×.

#### 3.5.5. EA

The samples were burned in an oxygen flow, and the organic components were fully oxidized with oxidants so that various elements were quantitatively transformed into their corresponding volatile oxides. These products were then made to flow through the silica gel packed column chromatography, and the thermal conductivity cell detector determined their concentrations. Finally, the content of each element was determined by the standard external method.

## 4. Conclusions

In this investigation, the sugar yield from BW-pretreated samples was 2.07 times higher than that of untreated MDF, with a nitrogen removal rate of 58.91%. This underscores the efficacy of removing adhesives and additives in augmenting the enzymatic reduction of sugar yields from waste MDF. For the first time, waste MDF has been employed for biomass energy conversion. This study explored a cost-efficient, dual-step NaOH and IL pretreatment method for waste MDF, which not only effectively mitigated the impact of adhesives and additives but also facilitated a substantial glucose yield of 445.5 mg/g within 72 h. Compositional analysis, FT-IR, and ^13^C NMR revealed the extensive removal of lignin and hemicellulose during the pretreatment phase. The XRD analysis indicated an increase in the relative CrI of MDF following NaOH and AAI pretreatment, suggesting the removal or dissolution of some amorphous cellulose components. The SEM results demonstrated that both NaOH and AAI pretreatments effectively disrupted the surface structure of MDF. These analytical findings contribute valuable insights for further exploration into the energy conversion of waste fiberboard post-pretreatment and provide a robust foundation for subsequent investigations into the impact of material pretreatment on the outcomes of bioethanol production.

## Figures and Tables

**Table 1:** Effect of sodium hydroxide concentration and pretreatment time on reducing sugar yield.

Run	NaOH Aqueous (%)	Pretreatment Time (h)	Sugar Yield (mg/g)
1	1	1.0	350.28
2	3	1.0	360
3	5	1.0	378.27
4	1	1.5	338.04
5	3	1.5	347.85
6	5	1.5	363.15
7	1	2.0	278.19
8	3	2.0	294.03
9	5	2.0	324.36

**Table 2:** Sugar yield of raw MDF, hot-mill MDF, and pretreated MDF samples.

Samples		Sugar Yield (mg/g)
Eucalyptus	Raw	193.2
	Hydrothermal	205.4
Hot mill MDF	Raw	237.8
	Hydrothermal	248.8
MDF	Raw	88.5
	Hydrothermal	167.6

**Table 3:** Sugar yield and elemental analysis of each sample.

Pretreatment Methods	Sugar Yield (mg/g)	N [%]	C [%]	H [%]	S [%]
MWI	281.3	3.96	45.58	6.842	0.051
BW	183.2	1.89	45.56	6.896	0.022
BW + IL	406.1	1.54	46.24	6.761	0.058
MDF	88.5	4.60	44.69	6.791	0.000
Eucalyptus	193.2	0.95	45.35	7.074	0.000

**Table 4:** Chemical compositions of raw MDF and NaOH- and AAI-pretreated MDF samples.

Pretreatment Methods	Solid Recovery (%)	Glucan (%)		Xylan (%)		Lignin (%)	Ash (%)
		——	Enzymatic Hydrolysis Yield	——	Enzymatic Hydrolysis Yield		
Untreated	——	38.18 ± 0.52	23.28	27.45 ± 0.17	27.25	27.35 ± 0.85	0.89
NaOH	60.9 ± 1.0	48.91 ± 0.2	77.34	18.93 ± 0.44	43.21	23.67 ± 0.41	1.48
AAI	57.6 ± 1.1	53.4 ± 0.14	83.42	16.14 ± 0.21	51.74	21.4 ± 0.31	1.32

**Table 5:** The characteristic peaks and attributions of the outer spectrum of wood pink.

Wave Number (cm^−1^)	Spectral Peak Attribution Analysis
1740	Carboxyl group, carbonyl group, and acetyl group in hemicellulose C=O stretching vibration.
1596	Benzene ring skeleton stretching vibration plus C-O stretching vibration.
1515	C=C stretching vibration of aromatic ring skeleton in lignin.
1355	Vibration of cellulose and hemicellulose C-H.
1268	C-H stretching vibration in cellulose, C-O stretching vibration in syringa.
1244	Stretching vibration of C-O in hemicellulose or lignin.
898	C-H deformation vibration of β-glycosidic bond.

## Data Availability

All data generated or analyzed during this study are included in this published article.
